# Training in Complementary Feeding Counselling of Healthcare Workers and Its Influence on Maternal Behaviours and Child Growth: A Cluster-randomized Controlled Trial in Lahore, Pakistan

**Published:** 2008-06

**Authors:** Shakila Zaman, Rifat N. Ashraf, José Martines

**Affiliations:** ^1^Health Services Academy, Islamabad, Pakistan; ^2^Department of Social and Preventive Paediatrics, King Edward Medical College, Lahore, Pakistan; ^3^Department of Child and Adolescent Health, World Health Organization, 1211 Geneva 27, Switzerland

**Keywords:** Child, Child-feeding practices, Child growth, Child nutrition disorders, IMCI, Impact studies, Infant, Infant growth, Infant nutrition disorders, Nutrition counselling, Randomized controlled trials, Training, Pakistan

## Abstract

Malnutrition is common among children aged 6–24 months in developing countries. It increases the risk of mortality. Interventions to improve infant-feeding hold the promise of reducing malnutrition among these children. A study in Brazil has shown the success of training in communication and counselling skills among health workers in improving the nutritional status of young children. Questions were raised whether the method used in the study in Brazil would also be effective when applied in other countries. The aim of the present study was to reduce growth faltering in young children through proper nutrition-promotion techniques. The objective of the study was to determine the efficacy of training health workers in nutrition counselling in enhancing their communication skills and performance, improving feeding practices, and reducing growth faltering in children aged 6–24 months. A cluster-randomized controlled trial was carried out. The method used in this study was a replica of the method in a similar study in Pelotas, Brazil. Forty health centres were paired, and one centre of each pair was randomly allocated to the intervention group, and the other to the control group. The Integrated Management of Childhood Illness (IMCI) module—‘Counsel the mother'—was used for training health workers in the health centres in the intervention group. Data from 36 paired health centres and 375 mothers and their children aged 6–24 months recruited from these health centres following consultation with health workers were included in analysis. Independent observers, masked to the intervention status, examined the performance of health workers within the first month after training. Mother-child pairs were visited at home within two weeks, 45 days, and 180 days after recruitment. Information was recorded on the feeding practices, recall of the recommendations of health workers, and sociodemographic variables at these home-visits. Weight and length of the child were measured at each contact. The communication skills and consultation performance of health workers were significantly better in the intervention group than in the control group. The mothers' recall of the recommendation of health workers and reported infant-feeding practices were also significantly better in the intervention group than in the control group, even 180 days after the recruitment consultation. Growth faltering was less in the intervention group, with the largest effect observed among children in the age-group of 12 + months. These results indicate that training in IMCI feeding counselling can enhance the communication skills and performance of health workers. Improved feeding practices of counselled mothers can, in turn, reduce growth faltering in their children.

## INTRODUCTION

Promotion of appropriate feeding practices is fundamentally important in reducing child malnutrition and mortality (1) and, thus, for achieving Millennium Development Goals 1 and 4. Counselling has been shown to increase knowledge of caregivers and to improve breastfeeding, complementary feeding, and growth in young children (2-5).

Consultations for the care of sick children present a major opportunity for the delivery of feeding counselling to mothers. Questions have been raised, however, on how effective the use of this contact opportunity would be in promoting improved feeding and growth. A study conducted in Brazil to test the efficacy of training health workers to counsel caregivers on the feeding of young children during consultations when the child was sick indicated that it can improve feeding practices and gains in weight among children aged 12 months or older (6). The authors recognized the need to replicate the study in settings where (a) there is a higher prevalence of malnutrition, (b) the duration of breastfeeding is longer, (c) maternal education is lower, and (d) the availability of food is more likely to be a constraint. The site chosen for this study in Lahore, Pakistan, presents the type of situation re-commended for replication (7-8). In this setting, we tested again the hypothesis that training first-level health workers for child-feeding counselling would lead to a reduction in growth faltering during complementary feeding. We examined the following steps: whether training health workers increased the provision of adequate feeding counselling to caregivers; whether this, in turn, improved the knowledge and practices of caregivers for child-feeding; and, finally, whether these improved feeding practices were associated with a reduction in growth faltering.

This study aimed at reducing growth faltering in young children through proper nutrition-promotion techniques. The objective was to determine the efficacy of training health workers in nutrition counselling in enhancing their communication skills and performance, improving feeding practices, and reducing growth faltering in children aged 6–24 months. A cluster-randomized controlled trial was carried out.

## MATERIALS AND METHODS

### Study design and sample selection

The study was designed as a single-blind randomi-zed controlled trial. Of the 60 healthcare centres operated by the Directorate of Health and the Lahore Metropolitan Corporation (LMC), 40 were selected for the study. They were paired by the category of healthcare provider, by complexity of the healthcare facility, and by the type of area they served. One member of each of 20 pairs was allocated to the intervention group and the other to the control group randomly by flipping a coin.

### Sample size

Two major outcomes were considered in calculating the sample size: the proportion of healthcare providers with adequate nutritional counselling performance (for instance, who recommended changes in inappropriate feeding practices) and in the average improvement in nutritional status in a six-month period. The level of significance was set at 5% (one-tailed test) and the statistical power at 90%. Assuming that 40% of health workers in the control group provide adequate counselling with a range from 20% to 60% across different health centres, and if three health workers were assessed in each health centre, 18 health centres in each group would allow adequate study power to detect an increase in adequate counselling performance to 75% in the intervention group, after adjusting for cluster randomization. Assuming the mean (standard deviation [SD]) weight-for-age z-scores of children in the control group to be −1.5 (SD 1.2) and a design effect of 1.5, the same number of health centres would be sufficient to detect a lower mean weight-for-age z-score of −1.0 if 10 infants were recruited in each health centre. We aimed to include 20 health centres in each group to allow a margin of 10% for possible losses.

### Study groups

In each health centre, a Lady Health Visitor (LHV) is responsible for the management of sick mothers and children reporting to her centre. All LHVs of the intervention centres were trained in nutrition counselling using the module—‘Counsel the Mother'—of the World Health Organization (WHO)/United Nations Children's Fund IMCI training course (9). The training lasted for five half-days and included updating infant-feeding knowledge and practice sessions for the development of communication and counselling skills. A local adaptation of Pakistan's IMCI ‘feeding counselling card' was developed in the local language. The card was intended to assist the LHVs in the process of counselling, highlighting the recommended foods and frequency of feeding to be discussed with mothers according to age of the child and to act as a reminder at home of the recommendations received at the health centre.

#### Field methodology

An independently-trained group of field workers conducted structured observations in at least two consultations at each of the health centres within the first month after training.

At the same time, the trained health worker recruited the target groups (children to participate in this study) after obtaining informed consent from the mothers. For operational reasons, only the age, identification and address, and data required for locating the family in the community were collected at the time of recruitment. The first 10 children aged 6–18 months, coming for consultation at each health centre, were selected. Any child reporting with illness for which referral was required was excluded. Any child not living in the study area or who was reportedly expected to move away during the following six months was also excluded.

### Outcome measures

A separate team of observers who were blind to the intervention or control status of the LHVs, mothers, and children collected information on the study outcomes. Information included observations of performance of LHVs during consultation in terms of assessment of feeding, provision of recommendations, and their counselling skills; maternal recall of child-feeding recommendations; reported child-feeding practices; and measured weight and length. These data were collected during visits to home conducted within two weeks, 45 days, and 180 days after recruitment. At each home-visit, ownership of the ‘feeding counselling' card and understanding of its contents were checked with the mothers in the intervention group.

**Fig. 1:**
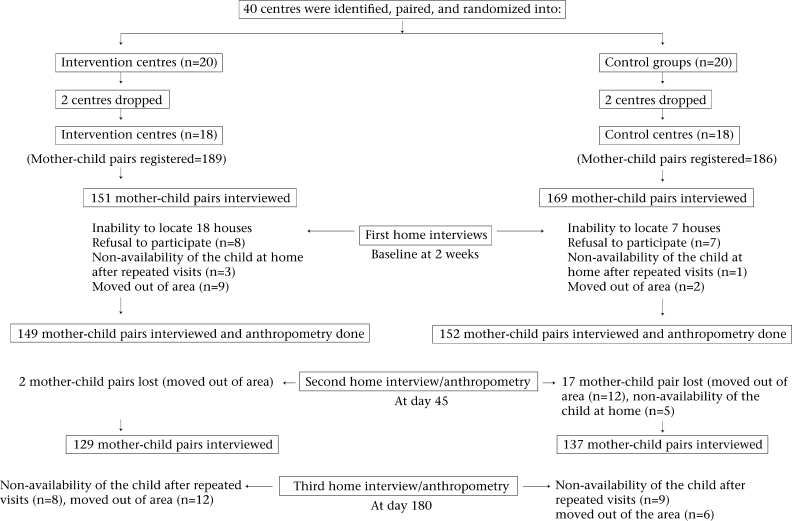
Trial profile

Four centres were dropped from the study after the initial allocation as either intervention (n=2) or controls groups (n=2) because their use was so low that it would not be operationally feasible to reach the target number of 10 mother/child pairs recruited within six months after training. The trial profile is presented as Figure 1.

### Data analysis

Data were entered using the EPI Info software (version 9.0) with automatic checks for range and consistency.

Data on weight, length, or height at all ages were converted into standard deviation (SD) scores using the WHO/National Center for Health Statistics reference as the standard population. Initial analysis of data was done using the SAS software (Version 9.0).

Initially, the intervention and control groups were compared in terms of baseline indicators, such as socioeconomic variables, age of children, sex, and educational status of parents. The intervention and control groups were then compared for outcome measures as described above using the Stata software (version 8.0). The level of significance was considered at 0.05 (10). Odds ratios and 95% confidence intervals (CIs) were also calculated to explore some relationships. Robust confidence intervals were used, adjusting for the cluster effect.

### Quality control

These measures included blinding of the observation team and their supervisors regarding the intervention or the control status of health workers, mothers, and children. Pretested, standardized questionnaires and detailed guides for interviewers were used for the interviews with careful selection and evaluation of interviewers and regular retraining, including thorough training on interviewing and anthropometric measurements, followed by standardization sessions with assessment of intra- and interobserver variability. A supervisor made weekly check-ups of the measuring instruments and repetition of 10% of the interviews and measurements. Simultaneous data entries with range and consistency checks were applied to the collected data.

### Ethical issues

The study complied with the international guidelines for biomedical research involving human subjects (11). The children in the control group continued to receive routine nutritional and health advice. Ethical approvals by the King Edward Medical College Ethics' Committee and by the Steering Committee on Research Involving Human Subjects of the WHO were obtained. Informed consent was obtained prior to the enrolment of LHVs, mothers, and children. This was done with a clear description of the objectives of the study and of its procedures and after ascertaining that this information had been adequately understood. Confidentiality of the information obtained from the healthcare providers, mothers, and children was strictly maintained. Children with severe disease or severe malnutrition were immediately referred to the hospital. Children found to be malnourished or ill during home-visits were referred to the nearest health centre for appropriate care.

## RESULTS

### Knowledge, practices, and counselling skills of healthcare providers

Table 1 shows the differences between the intervention and the control group observed during the consultations by the independent observers. The communication skills were significantly different in the two groups for making friendly remarks (82% vs 51%, p=0.0152), asking about feeding and paying attention to the answers (50% vs 25%, p=0.0561), and praising the mother for positive action (37% vs 8%, p=0.0059).

**Table 1:** Observed performance of health workers

Observations	Intervention group observations (n=52)		Control group observations (n=53)		Odds ratio	95% CI*	p value*
	No.	%	No.	%			
Communication skills							
Greets cordially	46	88.46	44	83.02	1.56	0.29–8.32	0.5972
Passes friendly remarks	43	82.69	27	50.94	4.60	1.32–15.92	0.0160
Pays attention to mothers	47	90.38	45	84.91	1.67	0.38–7.34	0.4980
Encourages mothers to talk	33	63.46	28	52.83	1.55	0.48–4.99	0.4624
Positive non-verbal communication and body language	49	94.23	48	90.57	1.70	0.28–10.51	0.5631
Asks about feeding and pays attention to reply	26	50.00	13	24.53	3.07	0.93–10.12	0.0643
Praises the mother for positive action	19	36.54	4	7.55	7.05	1.68–29.5	0.0075
Recommends changes in inappropriate feeding practices	17	32.69	2	3.77	12.38	2.43–63.25	0.0025
Explains why changes have to be done	15	28.85	2	3.77	10.34	2.05–52.18	0.0047
About feeding							
Asks if the child is breastfed	26	50.03	12	27.27	3.15	0.95–10.43	0.0602
Asks about other foods and drinks	24	46.15	6	11.76	6.42	1.37–30.1	0.0181
Asks size of the portion	14	27.45	3	5.66	6.18	1.04–36.6	0.0447
Asks if changed feeding during illness	7	15.56	4	9.09	2.00	0.46–8.73	0.3530
Actions							
Weighs the child	30	57.69	25	47.17	1.52	0.50–4.64	0.4555
Plots weight in growth chart	25	36.54	4	7.95	7.05	1.16–42.7	0.0335
Checks current feeding against age recommended feeding	21	38.46	3	5.66	10.4	1.91–56.8	0.0068
Checks if the mother has understood	15	29.41	1	1.89	21.66	2.6–181.93	0.0046

*Robust to account for cluster randomization; CI=Confidence interval

Regarding assessment questions about feeding, no differences reached the level of statistical significance. However, appropriate actions, such as recommending specific changes in inappropriate feeding practices and explaining why the changes should be made (29% vs 4%, p=0.01), were significantly more frequent in the intervention group than in the control group. The trained LHVs were also significantly more likely to plot the weight of the child, discuss the foods appropriate for the age of the child, and check if the mothers understood information provided.

### Sociodemographic information

The socioeconomic and demographic characteristics of the two groups were similar. Males and females were equally distributed among the two groups, the educational status of mothers did not differ significantly, and the anthropometric measurements were similar among the two groups at the start of the study (Table 2).

**Table 2:** Baseline sociodemographic characteristics and nutritional status of children enrolled in the study

Characteristics	Intervention group		Control group		p value*
	No.	%	No.	%	
Gender					
Male	83	54.97	84	49.70	
Female	68	45.03	85	50.30	0.4350
Age (months) of children					
6–9	34	22.5	45	26.6	
9–12	54	36.0	66	39.0	
12+	63	41.7	58	34.3	0.381
Total	151		169		
All ages together					
Mean weight/age z-score (SD)	−1.089 (1.22)		−1.439 (1.22)		0.125
Mean height/age z-score (SD)	−1.115 (1.36)		−1.407 (1.22)		0.167
Mean weight/height z-score (SD)	−0.450 (1.01)		−0.559 (1.08)		0.451
By age-groups					
6–9 months					
Mean weight/age z-score (SD)	−0.7144 (1.05)		−1.453 (1.69)		0.102
Mean height/age z-score (SD)	−0.609 (1.33)		−1.44 (1.45)		0.067
Weight/height z-score (SD)	−0.366 (0.89)		−0.582 (1.52)		0.485
9–12 months					
Weight/age z-score (SD)	−0.987 (1.36)		−1.408 (1.07)		0.217
Height/age z-score (SD)	−1.040 (1.33)		−1.355 (1.17)		0.364
Weight/height z-score (SD)	−0.287 (1.10)		−0.713 (1.12)		0.104
12+ months					
Weight/age z-score (SD)	−1.379 (1.15)		−1.46 (0.92)		0.644
Height/age z-score (SD)	−1.44 (1.34)		−1.55 (1.08)		0.559
Weight/height z-score (SD)	−0.34 (0.95)		−0.81 (1.02)		0.048
Mothers' level of schooling					
No schooling/illiterate	53	35.10	78	42.96	0.3571
1–5 year(s)	16	10.60	18	10.65	0.6795
6–12 years	56	37.08	58	34.43	0.6705
12 years and more	26	17.22	15	8.88	0.8122

*Robust to account for cluster randomization; SD=Standard deviation

### Recall by mothers

Table 3 presents mothers' recall of the advice of health workers on child feeding. Mothers were asked whether they remembered any feeding advice given by the LHVs during their visits to the clinic. Spontaneous and prompted recall information was recorded. At first contact at home, within two weeks after the training, the recall of correct feeding information was significantly higher in the intervention group than in the control group. One hundred and eighty days later, the recall was still significantly higher in the intervention group than in the control group. Mothers belonging to the intervention group were 1.5–3 times more likely to recall correct advice delivered during the consultation.

**Table 3:** Recall of health worker's advice: mother's recall of advice on feeding that was given to her during visits to the clinic 8 days to 2 weeks and 180 days after recruitment

Foods recommended	Intervention group (n=151)		Control group (n=169)		Odds ratio	95% CI*	p value*
	No.	%	No.	%			
After 8 days–2 weeks							
Use cup and spoon instead of a bottle	83	54.97	52	30.77	2.74	1.74–4.34	0.0373
Increase the amount of milk offered	58	38.41	41	24.26	1.95	1.20–3.15	0.0352
Increase the variety of foods	93	61.59	60	35.5	2.92	1.84–4.59	0.0039
Increase the density of porridge	85	56.29	69	40.83	1.86	1.19–2.90	0.0025
Offer eggs	86	56.95	69	41.53	1.92	1.20–2.99	0.0034
Add oil/ghee/butter to the child's food	39	25.83	22	13.0	2.33	1.36–4.14	0.0257
Give poultry/beef/lamb/fish mashed/small pieces	86	56.95	58	32.34	2.55	1.61–3.8	0.0028
Give liver	33	21.8	21	12.49	1.98	1.08–3.58	0.0131
Add green-leafy vegetables	68	45.03	49	28.99	2.00	1.30–3.18	0.0156
After 180 days							
Use cup and spoon instead of a bottle	78	51.66	49	29.17	2.59	1.64–4.11	0.0393
Increase the amount of milk offered	73	48.0	49	29.2	2.28	1.43–3.60	0.0324
Increase the variety of foods	87	57.62	58	34.50	2.57	1.64–9.05	0.0405
Increase the density of porridget	96	63.58	66	39.30	2.69	1.71–4.25	0.0343
Offer eggs	98	64.90	64	38.10	3.00	1.90–4.70	0.0569
Add oil/ghee/butter to the child's food	53	35.10	29	17.26	2.59	1.54–4.37	0.0447
Give poultry/beef/lamb/fish mashed/small pieces	96	63.58	63	37.52	2.90	1.84–4.58	0.0329
Give liver	54	35.76	36	21.93	2.04	1.24–3.35	0.0536
Add green-leafy vegetables	91	60.26	57	33.03	2.95	1.88–2.7	0.0490

*Robust to account for cluster randomization; CI=Confidence interval

### Reported feeding practices and effect of recommendations of health workers

Table 4 presents the feeding practices reported by mothers and the responses to the recommendations of health workers in terms of mothers offering specific foods. Within two weeks of consultation, the proportion of mothers reporting to offer eggs and meat to their children was significantly higher in the intervention group (p=0.03 and 0.01) than in the control group. Feeding practices tended to improve in both the groups as children grew older. For example, while the consumption of liver increased from 17% to 31% in the intervention group between two weeks and 180 days, it also increased from 9.5% to 20% in the control group during the same period. However, a significantly greater proportion of children in the intervention group was offered eggs (p=0.043) and thick *kitchuri* (p=0.044) even after 180 days.

**Table 4:** Feeding practices: reports by mothers of offering the child foods recommended by the health worker 8 days to 2 weeks and 180 days after recruitment

Reported practices	Intervention group		Control group		Odds ratio	95% CI*	p value*
	No.	%	No.	%			
8 days–2 weeks	(n=151		(n=169)				
Offered eggs	72	47.68	54	31.95	1.94	1.04–3.62	0.0368
Offered chicken/beef/mutton	75	49.67	54	31.95	2.10	1.15–3.83	0.0155
Offered liver	26	17.22	16	9.47	1.99	0.89–4.44	0.0934
Added ghee/butter/oil	46	30.46	42	24.85	1.32	0.51–3.42	0.5616
Offered thick *kitchuri*	93	61.59	76	44.97	1.96	0.95–4.05	0.0682
180 days	(n=126)		(n=131)				
Offered eggs	60	47.62	35	26.72	2.49	1.03–6.03	0.0428
Offered chicken/beef/mutton	76	60.32	52	39.69	2.30	0.996–5.34	0.0508
Offered liver	39	30.95	26	19.85	1.81	0.79–4.10	0.1589
Added ghee/butter/oil	68	53.97	50	38.17	1.89	0.75–4.78	0.1736
Offered thick *kitchuri*	83	65.87	58	44.27	2.43	1.02–5.76	0.0441

*Robust to account for cluster randomization; CI=Confidence interval

### Weight and length

**Fig. 2:**
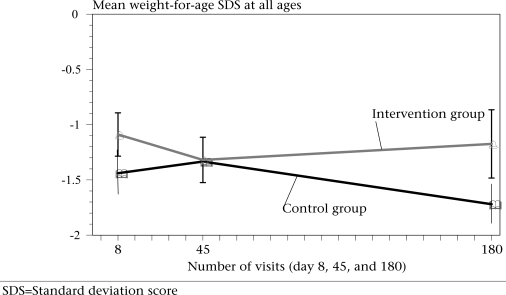
Shows the changes in the mean weight-for-age SDS by the number of visits at day 8, 45, and 180. All the ages are put together

The SD scores for weight and length are presented in Table 5. The mean SD scores of the two groups at the time of the first home-visit (8–15 days after recruitment) and at the time of the second home-visit were not significantly different. However, by the third visit on the 180^th^ day, the mean SD score for weight-for-age was significantly higher in the intervention group (−1.1742 vs −1.720 with p=0.012) (Fig. 2). There were no significant differences between the groups in the mean SD scores for length-for-age at any of the three visits.

**Table 5:** Mean weight-for-age z-score, height-for-age z-score, and weight-for-height z-score are expressed by age- groups and number of visits

Z-score	Intervention group			Control group			p value*
	No.	Mean	SD	No.	Mean	SD	
Total number of children by number of visits							
WAZ 1^st^ visit	171	−1.089	1.23	172	−1.439	1.22	0.125
WAZ 2^nd^ visit	168	−1.319	1.29	141	−1.334	1.19	0.950
WAZ 3^rd^ visit	143	−1.174	1.94	143	−1.720	1.27	0.012
HAZ 1^st^ visit	170	−1.115	1.36	172	−1.407	1.22	0.167
HAZ 2^nd^ visit	168	−1.360	1.29	141	−1.575	1.44	0.241
HAZ 3^rd^ visit	142	−1.582	1.58	142	−1.705	1.24	0.559
WHZ 1^st^ visit	170	−0.450	1.01	172	−0.559	1.08	0.452
WHZ 2^nd^ visit	168	−0.536	1.22	141	−0.382	1.08	0.447
WHZ 3^rd^ visit	142	−0.286	1.22	142	−0.794	1.15	0.0046
By age and number of visits 6–9 months							
WAZ 1^st^ visit	41	−0.7144	1.04	39	−1.453	1.69	0.102
WAZ 2^nd^ visit	35	−1.087	1.16	37	−1.361	1.36	0.404
WAZ 3^rd^ visit	35	−1.152	0.89	37	−1.863	1.56	0.067
HAZ 1^st^ visit	40	0.609	1.32	39	−1.44	1.45	0.067
HAZ 2^nd^ visit	35	−0.843	1.19	37	−1.64	1.19	0.087
HAZ 3^rd^ visit	35	−1.1489	1.31	37	−1.55	1.59	0.790
WHZ 1^st^ visit	40	−0.366	0.88	39	−0.582	1.525	0.485
WHZ 2^nd^ visit	35	−1.360	1.29	37	−1.575	1.172	0.988
WHZ 3^rd^ visit	35	−1.582	1.58	37	−1.705	1.344	0.048
9–12 months							
WAZ 1^st^ visit	61	−0.9868	1.36	55	−1.408	1.07	0.217
WAZ 2^nd^ visit	52	−1.231	1.46	55	−1.26	1.26	0.936
WAZ 3^rd^ visit	52	−1.22	1.22	53	−1.68	1.27	0.204
HAZ 1^st^ visit	61	−1.040	1.36	54	−1.355	1.17	0.364
HAZ 2^nd^ visit	52	−1.367	1.23	54	−1.557	1.13	0.564
HAZ 3^rd^ visit	52	−1.65	1.03	52	−1.676	1.15	0.932
WHZ 1^st^ visit	61	−0.287	1.10	54	−0.469	0.89	0.396
WHZ 2^nd^ visit	52	−0.352	1.31	54	−0.212	1.04	0.170
WHZ 3^rd^ visit	52	−0.272	1.29	52	−0.713	1.12	0.1043
12+ months							
WAZ 1^st^ visit	72	−1.379	1.15	62	−1.463	0.96	0.644
WAZ 2^nd^ visit	62	−1.52	1.19	51	−1.392	1.00	0.589
WAZ 3^rd^ visit	62	−1.143	0.97	51	−1.647	10.02	0.022
HAZ 1^st^ visit	72	−1.44	1.34	62	−1.55	1.09	0.559
HAZ 2^nd^ visit	62	−1.63	1.35	51	−1.82	1.09	0.377
HAZ 3^rd^ visit	62	−1.573	1.092	51	−1.82	0.87	0.171
WHZ 1^st^ visit	72	−0.634	0.98	62	−0.656	0.865	0.952
WHZ 2^nd^ visit	62	−0.73	1.19	51	−0.456	1.035	0.312
WHZ 3^rd^ visit	62	−0.35	0.947	51	−0.814	1.02	0.048

*Robust to account for cluster randomization; HAZ=Height-for-age z-score; SD=Standard deviation; WAZ=Weight-for-age z-score; WHZ=Weight-for-height z-score

**Fig. 3:**
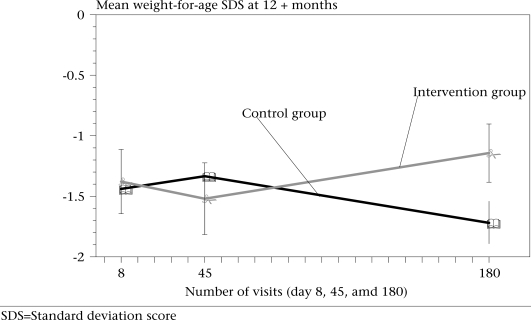
Shows the changes in the mean weight-for-age SDS by the number of visits at day 8, 45, and 180. Error bars are also shown. Only age 12+ months is shown here

Figure 3 shows the mean weight-for-age scores for children aged 12 months and older at the time of recruitment. It shows that the weight-for-age of the intervention and control groups remained similar up to the visit on the 45^th^ day; however, the differences in growth thereafter led to a significantly higher weight-for-age score among children in the intervention group by the time of the third (180^th^ day) visit (−1.143 vs −1.647, p=0.022).

The differences in the mean height-for-age at all ages and visits were not statistically significant.

## DISCUSSION

More than 50% of childhood mortality is associated with malnutrition (12). The risk of malnutrition increases significantly during 6–24 months, known as the complementary feeding period (13). Suboptimal infant-feeding practices are a key determinant of this faltering in growth (14,15).

The results of this trial support the findings of an earlier study in Pelotas, Brazil, proving that feeding counselling can lead to the improvements in feeding practices required to reduce growth faltering and that training of health workers on feeding counselling is an effective approach to the delivery of this intervention during their consultations for sick children. The finding of this study indicated that nutritional counselling was associated with reduced growth faltering among children aged 12 months or older at the time of consultation (6).

The examination of the differences between the intervention and the control group along the pathway towards reducing growth faltering generated detailed information on the adequacy of the intervention and strengthened the plausibility of the attribution of observed changes in child nutrition to feeding counselling training (16).

We first examined whether training would improve counselling and communication skills of health workers. Their communication and counselling skills were significantly improved after the training and health workers proved to apply these new skills in their practice during the weeks after training. A similar effect of training in feeding counselling using the IMCI materials was observed in Brazil (6). We observed, as well, that the adequacy of the content of the feeding recommendations provided by trained health workers was significantly improved due to the training. In Brazil, Pelto *et al*. observed a similar effect (3). The results showed that the caregivers who were counselled by trained health workers recalled more information provided during consultation, even six months after the training of health workers and recruitment of the child into the study. The responses, however, were different for the time of visits and also for the recall of the specific advice provided by the health workers. This indicates that the feeding practices were changed according to the ages, but within the context of the accepted practices among the mothers since the pattern is more or less consistent within the two groups.

The mothers in the intervention group were signifi-cantly more likely to report feeding their children with the recommended foods. This presumably resulted in a diet that was of improved quality in micronutrient content, in addition to the improvements in energy density expected from mothers in the intervention group feeding complementary foods of increased consistency (17).

Children in the intervention group experienced less growth faltering than the control children. Six months after the delivery of the intervention, their weight was significantly higher than that of the control group. The length-for-age was also higher, although the difference was not significant. The lack of a significant effect on linear growth may be due to the relatively short duration of the trial. Since weight responds faster to intervention than length, a longer time period might have been required to observe detectable differences (18).

The fact that this was a randomized study with blind evaluation, the outcomes strongly support a causal link. Due to the characteristics of the study, it was not possible to carry a double-blind trial. In interpreting the findings of the study, it is important, nonetheless, to keep in mind some limitations of the study. The first assessment of feeding practices and collection of anthropometric data occurred not at recruitment but two weeks after enrollment. The differences between the groups at this time could be at least partly due to an effect of the intervention, but they could be also partly due to real pre-existing baseline differences. The baseline differences, despite randomization and similarities in socioeconomic and demographic variables, could have accounted for some differences in outcomes in the intervention and control groups. However, the trial profile indicated that, initially, the loss to follow-up was greater in the control group compared to the intervention group (2 vs 17 pairs). We could not find any specific reason for the differential losses in the follow-up at the time of the first visit, i.e. eight days—two weeks after the recruitment.

Some factors may have contributed to the success of the intervention in addition to the training course. The generic nutrition-counselling guidelines were adapted to the Pakistani environment. Counselling cards presenting a selection of recommended local foods were explained to mothers to act as reminders for the recommendations and also to facilitate the sharing of information with other family members. The literacy rate among mothers in the two groups was not significantly different at any level of education. However, there was a small difference in the proportion of illiterate mothers in the control group compared to those in the intervention group. This might have caused the higher recall scores among mothers in the intervention group, but there were no significant differences in the analysis when the factor—education—was controlled.

The effectiveness of counselling interventions has been demonstrated in both developed and developing settings to improve breastfeeding practices with consequent benefits in reduction in morbidi-ty (2-3,16,19-20). The success of interventions to improve child nutrition through counselling for improved complementary feeding practices with continued breastfeeding has been less well-demonstrated (21). Information from this study indicates that feeding counselling delivered by trained health workers can reduce growth faltering.

Although significant, the effects of the intervention, nonetheless, were not sufficient to eliminate growth faltering. Some limitations of the intervention were: (a) despite their better counselling performance, only 58% of the trained health workers presented adequate performance in observed consultations when specific actions were observed; (b) feeding of some promoted foods, particularly chicken liver that would have been the richest source of zinc and iron in the available diet, was increased but still quite low; (c) the contact of mothers in the intervention group was in most cases restricted to a single counselling session (encounter). Other factors, such as high prevalence of diseases in the study site, particularly respiratory tract infections and diarrhoea, countered the effect of the intervention and added to the reasons for growth faltering among these young children (22,23).

Although counselling on complementary feeding had a significant impact on those children seeking care/treatment at a health centre, additional interventions are required to further reduce the faltering in growth during complementary feeding. In addition to continuous improvement of the performance of health workers, skills in community mobilization or in the use of community or mass media should be improved as well to increase the coverage of mothers giving proper feeding to their children, in line with the health messages promoted by the healthcare providers (24).
